# Transposable Element Tissue-Specific Response to Temperature Stress in the Stenothermal Fish *Puntius tetrazona*

**DOI:** 10.3390/ani13010001

**Published:** 2022-12-20

**Authors:** Elisa Carotti, Federica Carducci, Adriana Canapa, Marco Barucca, Maria Assunta Biscotti

**Affiliations:** Dipartimento di Scienze della Vita e dell’Ambiente, Università Politecnica delle Marche, Via Brecce Bianche, 60131 Ancona, Italy

**Keywords:** transposable elements, fish, silencing mechanisms, stress temperature, tissue-specific response, phenotypic plasticity

## Abstract

**Simple Summary:**

Teleosts are one of the most diversified group of vertebrates, as they colonized different aquatic environments worldwide. Stenothermal fish are characterized by a limited ability to tolerate temperature changes, therefore variations in this abiotic factor represent a threat for their survival. In the present study, we selected the tiger barb *Puntius tetrazona*, for which RNA-Seq data were available for brain, gill, and liver, to investigate the possible role of the transcriptional activity of transposable elements (TEs) in the rapid adaptation of this species. Our findings highlighted a tissue-specific response of TEs with a remarkable increase at 13 °C recorded in liver, strengthening the view of TEs as source of genetic variability acting positively in species resilience.

**Abstract:**

Ray-finned fish represent a very interesting group of vertebrates comprising a variety of organisms living in different aquatic environments worldwide. In the case of stenothermal fish, thermal fluctuations are poorly tolerated, thus ambient temperature represents a critical factor. In this paper, we considered the tiger barb *Puntius tetrazona*, a freshwater fish belonging to the family Cyprinidae, living at 21–28 °C. We analyzed the available RNA-Seq data obtained from specimens exposed at 27 °C and 13 °C to investigate the transcriptional activity of transposable elements (TEs) and genes encoding for proteins involved in their silencing in the brain, gill, and liver. TEs are one of the tools generating genetic variability that underlies biological evolution, useful for organisms to adapt to environmental changes. Our findings highlighted a different response of TEs in the three analyzed tissues. While in the brain and gill, no variation in TE transcriptional activity was observed, a remarkable increase at 13 °C was recorded in the liver. Moreover, the transcriptional analysis of genes encoding proteins involved in TE silencing such as heterochromatin formation, the NuRD complex, and the RISC complex (e.g., AGO and GW182 proteins) highlighted their activity in the hepatic tissue. Overall, our findings suggested that this tissue is a target organ for this kind of stress, since TE activation might regulate the expression of stress-induced genes, leading to a better response of the organism to temperature changes. Therefore, this view corroborates once again the idea of a potential role of TEs in organism rapid adaptation, hence representing a promising molecular tool for species resilience.

## 1. Introduction

The evolutionary success of a species is strictly related to their ability to cope with changes in environmental conditions. Phenotypic plasticity is the main mechanism used by organisms to respond to rapid environmental perturbations. Indeed, it can produce well-adapted phenotypes that, in turn, might improve the fitness of organisms [1,2]. Phenotypic plasticity is also the result of environmental sensitivity of the genome that, through modifications in the heterochromatin structure, varies the expression not only of genes but also of transposable elements (TEs) [3]. TEs are interspersed repeated sequences that constitute a considerable fraction in several eukaryotic genomes. They adopt different transposition mechanisms to move throughout the host genome and are classified as TEs of class I, also called retrotransposons, including Long Terminal Repeats (LTRs) and non-LTRs (LINE and SINE), which use an RNA intermediate molecule that is reverse transcribed into complementary DNA by a reverse transcriptase (RT), followed by the reintegration into the host genome through a copy and paste mechanism, and class II of TEs, also called DNA transposons, use a DNA intermediate molecule to transpose and are characterized by the cut and paste mechanism of transposition, with an exception made for Helitron and Maverick/Polinton, which mobilize by a self-synthesizing mechanism involving direct synthesis of a DNA copy [4]. TEs are molecular elements that are activated by biotic and abiotic factors, contributing to the flexible response of the genome to external stimuli. They can cause chromosome rearrangements, increase mutation rates, or influence the expression of nearby genes, triggering the rapid adaptation of organisms to new conditions [5,6,7,8]. Barbara McClintock (1984) [9] was the first to propose a helpful role of TE-induced mutations in response to stress. However, transposition events might have deleterious effects; hence, several protective mechanisms are turned on by the host genome for its safeguard. DNA methylation is the most widely adopted mechanism for silencing TEs and consists of the addition of methyl groups to DNA. Another epigenetic mechanism regulating TE transcription regards histone modifications mediated by the Nucleosome Remodeling and Deacetylase (NuRD) complex [10]. In mammals, this complex is recruited at the TE sequence level by Krüppel-associated box domain zinc finger proteins (KRAB-ZFPs) through the involvement of the KRAB-associated protein-1 (KAP1) co-factor. Although these two latter proteins are absent in actinopterygians, our group recently proposed a KRAB-like and tripartite motif containing 33 (TRIM33) proteins as substitutes for the NuRD complex functioning in this evolutionary lineage [11]. Another conserved mechanism of TE silencing is based on the action of Argonaute proteins (AGOs) that use small non-coding RNAs (ncRNAs), such as microRNAs (miRNAs) and short interference RNAs (siRNAs) [12,13]. AGOs, in association with members of trinucleotide repeat containing adaptor 6 (TNRC6), part of the GW182 protein family [14,15,16], form a large multiprotein complex known as RNA-Induced Silencing Complex (RISC) that performs a post-translational regulation through RNA degradation and/or translation inhibition [17,18].

While a relationship between TEs and environment has been well established for plants [19,20,21,22,23,24,25,26] and *Drosophila* [27], this issue has only recently been investigated in vertebrates [8,11,27,28,29,30]. Actinopterygii or ray-finned fish are a very diversified group adapted to aquatic environments worldwide, from salt to fresh waters, from cold to warm seas, and from high-elevation mountain lakes to extreme sea depths. Studies performed to date in this taxon have provided evidence that the evolution of a specific TE type is the result of an intricate relationship with the environment. Rhee and colleagues (2017) [31] reported a high content of RC/helitrons that might be responsible for the high genetic diversity observed in *Kryptolebias marmoratus* populations. Moreover, they have found a maintenance of the TE genomic content in the self-fertilizing hermaphroditic *K. marmoratus*. This feature seems to promote genome recombination that, in addition to a mixed mating strategy, might have contributed to the evolutionary adaptation to ecological pressure of a species. Analyzing 52 fish species, Yuan and co-workers (2018) [32] evidenced a positive correlation between the presence of specific repetitive elements and the aquatic environments in which the considered species live. The chromosomal diversification and the consequent rapid speciation of the Antarctic teleosts belonging to the genus *Trematomus* have been attributed to the TE mobilization of *DIctyostelium* Repetitive Sequence 1 (DIRS1) [33]. Our research group performed a phylogenetic study evidencing an unexpected clusterization of the Rex3 retroelements in fish species living in cold environments independently from their taxonomic relationships [29]. Recently, we also published a paper concerning the possible correlation between the migratory behavior of catadromous teleost species and the amount of SINE retroelements [30]. In addition, analyzing RNA-seq data, we demonstrated for the first time an activation of TE transcriptional activity in response to salinity variations in the marbled eel *Anguilla marmorata* [11]. Since most teleosts are poikilotherms, environmental stimuli affect physiological and metabolic activities in these organisms [34,35,36,37]. In particular, ambient temperature might be a critical factor for stenothermal fish in which thermal fluctuations are less tolerated. The tiger barb *Puntius tetrazona* is a freshwater fish belonging to the family Cyprinidae, living at 21–28 °C. In this paper, we analyzed the RNA-Seq data of tiger barb exposed at 27 °C and 13 °C [38] to investigate the transcriptional activity of TEs and genes encoding for proteins involved in their silencing in the brain, gill, and liver. Our results pointed out a clear response of TEs in the liver consistent with the idea of a potential role of these elements in the rapid adaptation, thus representing a promising molecular tool for species resilience. Furthermore, all TE silencing mechanisms were active in the hepatic tissue and *ago* genes also in the brain and gill tissues.

## 2. Materials and Methods

Tiger barb RNA-Seq raw data were obtained from the Sequence Read Archive (SRA) (https://www.ncbi.nlm.nih.gov/sra, accessed on 15 June 2022) under the accession number SRP153005 [38]. Data were obtained sequencing the brain, gill, and liver of ninety fish (eight-month-old) divided into two groups, one used as the control group (27 °C) and another for testing the acute cold stress until 13 °C. The temperature was set to 27 ± 0.5 °C on the first day, followed by a decrease of 2 °C every 24 h. Fish were kept at 13 ± 0.5 °C for 24 h, and then, five fish, in triplicate, in each tank, were anesthetized before dissection. Raw paired-end reads were imported in the CLC Genomics Workbench v.12 (Qiagen, Hilden, Germany) (Table S1) and trimmed removing sequencing adapters, low-quality bases, and low-quality read ends using default parameters. Trimmed reads were then de novo assembled with the “De Novo Assembly” of CLC Genomics Workbench v.12 using the default parameters. Completeness of the de novo assembled transcriptome was evaluated through BUSCO v.5 using the Actinopterygii OrthoDB v.10 database as a reference [39].

### 2.1. Estimation of TE Transcriptional Activity

To estimate the TE transcriptional activity, we first identified TEs in the de novo assembled transcriptomes with RepeatMasker v.4.1.0 (http://www.repeatmasker.org/cgi-bin/WEBRepeatMasker, accessed on 15 July 2022) using a custom library created following the methodology described in our previous work [30]. After the RepeatMasker analysis, the trimmed reads related to 27 °C and 13 °C were mapped against the reference transcriptome to calculate the expression values using the proprietary RNA-Seq Analysis tool included in the CLC Genomics Workbench v.12 and setting the following mapping parameters: length fraction = 0.75 and similarity fraction = 0.98. To remove redundancies, the RepeatMasker output file was filtered, removing entries not classified as TEs and keeping those with the highest score and length values. The overall expression of each TE class was calculated by summing the expression values of each TE type: DNA transposons, LINE, LTR (also including endogenous retroviruses), non-LTR, Retro (retroelements that are not classified in any of the two main subclasses), and SINE. In our results, we also reported the fraction of unclear elements that are referred to repetitive elements without specific features to determine their attribution to a given TE class. We included this fraction to show the low percentage of the unclear fraction, representative of the goodness of our analyses. The expression values were then transformed into a percentage of mapped reads to achieve the comparability between species.

### 2.2. Identification and Expression Enalysis of Genes of Interest

Genes of interest were retrieved through TBLASTN [40] from the assembled transcriptome obtained from the brain, gill, and liver of *P. tetrazona*. In particular, the search was made for genes involved in heterochromatinization (*chromobox homolog 5* (*cbx5*), *chromobox homolog 1* (*cbx1*), *chromobox homolog 3* (*cbx3*), *DNA (cytosine-5-)-methyltransferase 1* (*dnmt1)*, *DNA (cytosine-5-)-methyltransferase 3 alpha* (*dnmt3a*)*,* and *SET domain bifurcated histone lysine methyltransferase 1* (*setdb1*)); genes related to the NuRD complex (*krab-like*, *trim33*, *chromodomain helicase DNA binding protein 3* (*chd3*), *chromodomain helicase DNA binding protein 4* (*chd4*), *histone deacetylase 1* (*hdac1*), *methyl-CpG binding domain protein 2* (*mbd2*), *methyl-CpG binding domain protein 3* (*mbd3*), *metastasis associated 1* (*mta1*), *metastasis associated 1 family, member 2* (*mta2*), *metastasis associated 1 family, member 3* (*mta3*), *GATA zinc finger domain containing 2* (*gatad2*), and *retinoblastoma binding protein 4* (*rbbp4*), *retinoblastoma binding protein 7* (*rbbp7*)); four genes of the *Argonaute* subfamily (*argonaute RISC component 1* (*ago1*), *argonaute RISC component 2* (*ago2*), *argonaute RISC component 3* (*ago3*), and *argonaute RISC component 4* (*ago4*)); and three genes of the GW182 family proteins (*tnrc6a*, *tnrc6b*, and *tnrc6c*). Transcripts were translated using the EMBOSS Transeq translation tool (https://www.ebi.ac.uk/Tools/st/emboss_transeq/, accessed on 12 September 2022), and the UTR and CDS regions were identified (Table S2). The aforementioned sequences were deposited in GenBank under the accession numbers listed in Table S2. To ensure the comparison between species, the gene expression values were computed using a scaling factor based on the cumulative expression of a dataset composed of 2124 orthologs derived from the Actinopterygii OrthoDB v.10 database [39]. In detail, the dataset was created as follows: for each transcriptome of the tissue considered in this study, expression levels of the genes attributed to the BUSCO analyses as “complete and single copy” and “fragmented” genes were kept as the number of mapped reads; expression values of genes classified as “duplicated” (most probably derived from transcriptional isoforms) were computed as the sum of each copy of single BUSCO, and the expression levels of “missing” genes were set to 0. This dataset was then used as a calibration set, computing a scaling factor that was applied to the original expression values of the genes of interest, as described in Biscotti et al. (2016) [41]. Transcriptional values of the genes analyzed in this study were reported as Transcripts Per Million (TPM).

### 2.3. Statistics 

For each tissue, the data on gene expressions and TE transcriptional activity obtained from the three replicates for each tested condition were expressed as the mean ± standard error, and statistically significant differences were evaluated by one-way ANOVA. The symbol * indicates *p*-values < 0.05, ** for *p*-values < 0.01, and *** for *p*-values < 0.001.

## 3. Results

**Figure 1:**
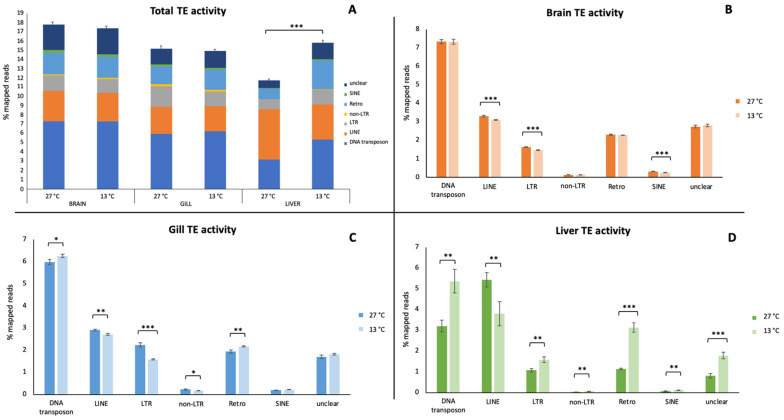
Transcriptional contribution of transposable elements in the brain, gill, and liver in *Puntius tetrazona*. (**A**) Total TE transcriptional contribution in the three analyzed tissues. (**B**) Transcriptional contribution of each TE type in the brain. (**C**) Transcriptional contribution of each TE type in the gill. (**D**) Transcriptional contribution of each TE type in the liver. Values expressed as the percentage of mapped reads are reported for both tested temperature conditions (27 °C and 13 °C) for each tissue. Statistically significant differences are presented as * for *p* < 0.05, ** for *p* < 0.01, and *** for *p* < 0.001.

The transcriptional activity of the TEs was evaluated as a percentage of the mapped reads in the brain, gill, and liver of the tiger barb (Figure 1). The data reported in Figure 1A were referred to the total TE transcriptional contribution at the control (27 °C) and test (13 °C) conditions. Comparing the total TE transcriptional activity at 27 °C between the analyzed tissues, the highest level was showed in the brain, followed by the gill and then liver. At 13 °C, the highest TE transcriptional activity in the brain was confirmed, and the difference between the gill and liver was less remarkable. Moreover, analyzing the data obtained in each tissue, a statistically significant difference between the two conditions considered here, was detectable only in the liver (*p*-value: 1.65 × 10^−5^), in which the total TE transcriptional activity was increased at 13 °C. Profiles of the TE relative abundances between the brain and gill samples were similar. Overall, a prevalence of DNA transposons followed by LINE, unclear, Retro, LTR, SINE, and non-LTR retroelements was shown. In the liver, LINE retroelements prevailed at 27 °C, while the TE distribution pattern was similar to that observed in the other two tissues at 13 °C. Despite the absence of an appreciable variation in the total contribution of TEs in the brain and gill, statistically significant differences emerged for the same tissues in single TE types between the two tested conditions. Indeed, in the brain, the LINE, LTR, and SINE retroelements showed a significant decrease passing from 27 °C to 13 °C (Figure 1B). In the gill, except for unclear elements and SINE retroelements, all TE types experienced significant changes, with DNA transposons and Retro having a higher activity at 13 °C, while LINE, LTR, and non-LTR retroelements have a lower activity in this condition than in the control one (Figure 1C). In the hepatic tissue, with the exception of LINE retroelements that showed lower values at 13 °C, all the analyzed TE types significantly increased their transcriptional contribution (Figure 1D). These increases were ascribable to a few specific elements. Indeed, the ten elements having the highest values of variation in transcriptional activity between the two conditions represented 62.27%, 86.0%, and 73.35% for the DNA transposons, SINE, and LTR retroelements, respectively. In particular, two elements, DNA/hAT-Ac and LTR/DIRS, mostly contributed to the upregulation of their belonging TE type (32.67% and 51.49%, respectively). Moreover, in the other tissues, the ten elements having the highest values of variation in transcriptional activity between the two conditions were different from those identified in the liver, except for one LTR and four SINE retroelements.

The transcriptional activity of TEs is controlled by the host genome through silencing mechanisms that determine a stronger heterochromatinization status through the activity of heterochromatin proteins, DNA methyltransferases, and the NuRD complex, and AGO proteins also involved in mRNA cleavage and translation repression. In this study, a total of 34 genes encoding proteins involved in TE silencing mechanisms were retrieved: ten concerning heterochromatin formation (*cbx5*, *cbx1a*, *cbx1b*, *cbx3a*, *cbx3b*, *dnmt1*, *dnmt3aa*, *dnmt3ab*, *setdb1a*, and *setdb1b*); 16 related to the NuRD complex (*krab-like*, *trim33*, *chd3*, *chd4a*, *chd4b*, *hdac1*, *mbd2*, *mbd3a*, *mbd3b*, *mta1*, *mta2*, *mta3*, *gatad2ab*, *gatad2b*, *rbbp4*, and *rbbp7*); four to *ago* (*ago1*, *ago2*, *ago3*, and *ago4*); and four belonging to the GW182 family proteins (*tnrc6a*, *tnrc6b*, *tnrc6c1*, and *tnrc6c2*). Of these, 20 showed a complete CDS, 2 were incomplete, and 12 fragmented gene sequences were replaced with the complete version available in public repositories to further refine the assembled transcriptome (Table S2).

**Figure 2:**
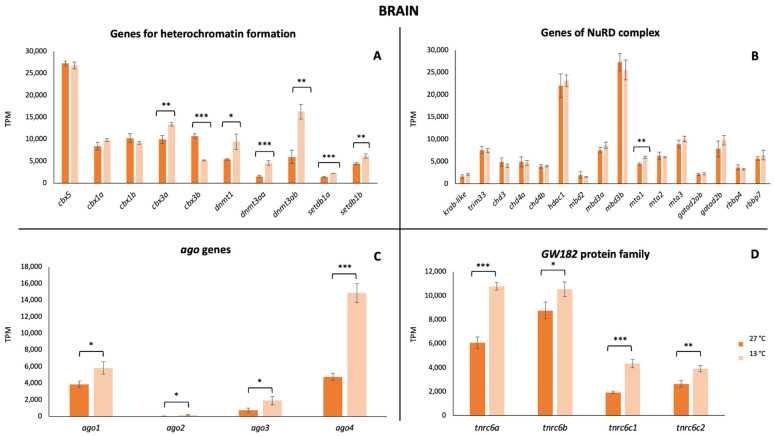
Expression of genes involved in TE silencing mechanisms in brain. (**A**) Expression values of genes encoding for proteins involved in heterochromatin formation. (**B**) Expression values of genes encoding for proteins forming the NuRD complex. (**C**) Expression values of members of the gene subfamily *Argonaute*. (**D**) Expression values of genes belonging to the GW182 family proteins. Values are reported for both tested temperature conditions (27 °C and 13 °C). Statistically significant differences are presented as * for *p* < 0.05, ** for *p* < 0.01, and *** for *p* < 0.001.

**Figure 3:**
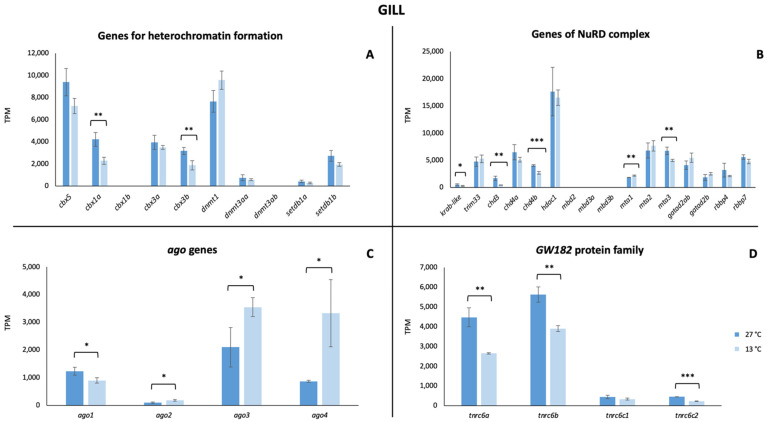
Expression genes involved in TE silencing mechanisms in the gill. (**A**) Expression values of genes encoding for the proteins involved in heterochromatin formation. (**B**) Expression values of genes encoding for proteins forming the NuRD complex. (**C**) Expression values of members of the gene subfamily *Argonaute*. (**D**) Expression values of genes belonging to the GW182 family proteins. Values are reported for both tested temperature conditions (27 °C and 13 °C). Statistically significant differences are presented as * for *p* < 0.05, ** for *p* < 0.01, and *** for *p* < 0.001.

**Figure 4:**
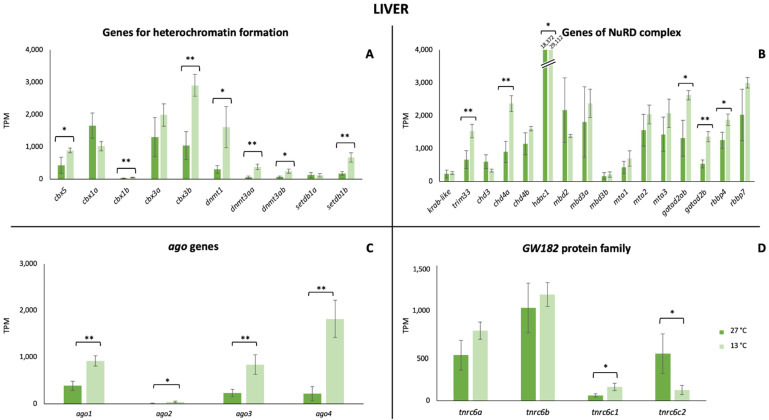
Expression genes involved in TE silencing mechanisms in the liver. (**A**) Expression values of genes encoding for proteins involved in heterochromatin formation. (**B**) Expression values of genes encoding for proteins forming the NuRD complex. (**C**) Expression values of members of the gene subfamily *Argonaute*. (**D**) Expression values of genes belonging to the GW182 family proteins. Values are reported for both the tested temperature conditions (27 °C and 13 °C). Statistically significant differences are presented as * for *p* < 0.05 and ** for *p* < 0.01.

Regarding the transcriptional activity, our findings pointed out that all these mechanisms were active in *P. tetrazona* (Figure 2, Figure 3 and Figure 4). In the case of the brain, a significant increase in the transcription of the genes involved in the heterochromatin formation was detected for *cbx3a,* as well as the three *dnmt* (*dnmt1*, *dnmt3aa*, and *dnmt3ab*), while a significant decrease was shown for *cbx3b* (Figure 2A). No remarkable change was detected comparing the expression of the genes encoding for the proteins of the NuRD complex between the two considered temperatures (Figure 2B) differently from the *ago* genes and those of the GW182 family proteins that recorded a statistically significant increase (Figure 2C,D). Analyzing the RNA-Seq data of a gill, a general statistically significant decrease in the transcriptional level of the genes involved in heterochromatin formation (*cbx1a*, *cbx3b)* and the NuRD complex (*krab-like*, *chd3*, *chd4b*, and *mta3*) was observed, while, in the latter, a significant increase was observed for *mta1* (Figure 3A,B). Differences in the expression of *ago* genes and in those encoding the GW182 family proteins were identified also in the gill (Figure 3C,D). In the hepatic tissue, most of the analyzed genes showed higher values at 13 °C (Figure 4). In particular, *cbx5*, *cbx1b*, *cbx3b*, *dnmt1*, *dnmt3aa*, *dnmt3ab*, *trim33*, *chd4a*, *hdac1*, *gatad2ab*, *gatad2b*, *rbbp4*, the four *ago* genes, and two of the GW182 family proteins showed significant changes.

## 4. Discussion

The ability of organisms to face changes of biotic and abiotic factors is mainly due to phenotypic plasticity that also requires modifications at the genome level. In particular, environmental changes can cause variations in the epigenetic status, leading to gene activation but also to the impairment of transposon silencing mechanisms inducing the activation of these mobile elements with the possible creation of genetic variability. Therefore, TEs represent a powerful adaptive response to environmental perturbations [7,8,27,42,43]. Although this issue has been largely studied in plants [20,21,22,25,44,45,46,47], information is still scarce in animals and, in particular, in vertebrates [27,43,48].

In this paper, we investigated the transcriptional TE response and the activity of genes involved in their silencing in the stenothermal fish *P. tetrazona*, a very popular ornamental freshwater fish native to Southeast Asia in places such as Malaysia and Borneo, places characterized by an equatorial climate; some of them are found in Thailand, Sumatra Island, and Cambodia [49]. This fish, living at 21–28 °C, tolerates small thermal fluctuations and experiences serious histopathological damages at low temperatures [38]. Overall, ambient temperature is an abiotic factor that acts on physiological and metabolic activities of poikilothermic teleosts, and in particular, on those of stenothermal fish [34,35,36,37]. Transcriptional analyses conducted by Liu and colleagues (2020) [38] on RNA-seq data obtained from *P. tetrazona* exposed at 27 °C (control) and 13 °C (test) have showed a high number of differentially expressed genes: in brain and liver the upregulated genes prevailed, differently from gill, in which the downregulated genes were the most represented. This is in line with the general idea that the genome epigenetic status varies in response to a stress, leading to changes in the expression levels. Our analysis of TE transcriptional contribution, performed on the same datasets, highlighted a different response in the three analyzed tissues. Apparently, the brain and gill showed the same behavior: in both the transcriptional activity of TEs did not vary between the two tested conditions. However, in the cerebral tissue, the activity of genes involved in heterochromatin formation, as well as *ago* genes and those related to GW182 family proteins, presented higher transcriptional levels at the stress condition. The lack of variation in TE transcriptional contribution in this tissue might be related to the silencing activity of these genes or to the short exposure period. In gill, although most of genes related to TE silencing mechanisms decreased their activity at 13 °C, no variation was observed in total TE transcriptional activity. However, for DNA transposons and Retro retroelements a slight increase of expression levels was appreciable at the stress condition. This might indicate that a chronic exposure is necessary to gain a remarkable TE variation. 

Interestingly, a statistically significant increase of TE transcriptional activity was showed only in liver when fish were exposed at 13 °C. Our findings revealed that this increase was mainly due to few specific TEs that seem to undergo a remarkable transcriptional variation only in the hepatic tissue. This is in line with previous papers, reporting a relationship between a kind of stress and a specific TE type response. The mPing DNA transposon has been reported to be activated in response to salt stress in rice [44,46], the ONSEN retrotransposon as consequence of heat stress in *Arabidopsis* [45,47], and the nuclear import of Tam3 transposon related-transposase has been showed to increase after exposition to temperature decrease in *Antirrhinum* [20,21,22,25]. In addition, the different TE behavior might be related to their tissue-specific response. Indeed, Hunter and colleagues (2012) [50] have reported a stress-induced activation of particular TE families as ERV intracisternal-A particle (IAP), B2_RN SINE, and L1_RN in the rat hippocampus.

It is well known that the transcriptional and transpositional activity of TEs can have positive effects increasing genetic variability and leading to advantageous phenotypes. On the other side, TEs can provoke negative effects, deleterious for the host genome that in turn activates TE silencing mechanisms. In the tiger barb *P. tetrazona*, the high TE transcriptional levels at 13 °C in liver were accompanied by an increase in the transcriptional activity of genes related to heterochromatinization, the NuRD complex, *ago*, and the GW182 family proteins. This picture might suggest that the hepatic tissue has a quicker responsiveness than other tissues. Indeed, it is a target organ for this kind of stress since several metabolic activities, also linked to body thermoregulation, occur in liver. Species respond to thermal conditions by modulating the metabolic rate to satisfy energy demand [51]. In *P. tetrazona* liver, Liu and colleagues (2020) [38] have reported an upregulation of cold-induced genes belonging to metabolism and biosynthesis pathways such as biosynthesis of steroid and fatty acids and tryptophan metabolism. The role of the hepatic tissue is extremely important since the tiger barb is a poikilothermic organism and also a stenothermal fish, tolerating small thermal fluctuations. The activation of pathways associated with organism response to stress conditions might be related to TE transcriptional activity. It is well-known that these genetic elements contribute in the up- or downregulation of nearby genes through *cis*- or *trans*-regulatory sequences located within TEs or TE-derived noncoding RNAs [26,52,53]. Alternatively, the decrease of methylation levels of genes involved in stress response might passively affect the heterochromatic state of nearby TEs leading to an increase of their transcriptional activity and consequently genes involved in their silencing are activated to counteract this epigenetic deregulation [54]. On the other side, liver might better tolerate the negative mutagenic effects related to TE activation thanks to its ability to regenerate after cell damage.

## 5. Conclusions

In conclusion, the analysis of the transcriptional activity of TEs and genes related to their silencing highlighted a clear response of these mobile elements in liver. Overall, the tissue-specific activation of TEs might represent a promising molecular tool, favoring species adaptation and resilience. Knowledge in this field could be useful in the perspective of wildlife species conservation since climate changes are threatening their biodiversity.

## Data Availability

Data are contained within the article or Supplementary Materials.

## Supplementary Materials

The following supporting information can be downloaded at: https://www.mdpi.com/article/10.3390/ani13010001/s1, Supplementary Table S1. Accession numbers of raw data downloaded from NCBI related to tested temperature conditions for brain, gill, and liver in *P. tetrazona*.; and Supplementary Table S2. Gene sequences analyzed in *P. tetrazona*. Details about the transcript length and its parts (5’ UTR, CDS, and 3’ UTR); aminoacidic length (aa); and information about its completeness and related accession numbers is reported.

## References

[1] West-Eberhard M.J. (2003). Developmental Plasticity and Evolution.

[2] Pigliucci M., Murren C.J., Schlichting C.D. (2006). Phenotypic plasticity and evolution by genetic assimilation. J. Exp. Biol..

[3] Feil R., Fraga M.F. (2012). Epigenetics and the environment: Emerging patterns and implications. Nat. Rev. Genet..

[4] Almojil D., Bourgeois Y., Falis M., Hariyani I., Wilcox J., Boissinot S. (2021). The structural, functional and evolutionary impact of transposable elements in eukaryotes. Genes.

[5] Kidwell M.G., Lisch D.R. (2001). Perspective: Transposable elements, parasitic DNA, and genome evolution. Evolution.

[6] Kazazian H.H. (2004). Mobile elements: Drivers of genome evolution. Science.

[7] Chénais B., Caruso A., Hiard S., Casse N. (2012). The impact of transposable elements on eukaryotic genomes: From genome size increase to genetic adaptation to stressful environments. Gene.

[8] Casacuberta E., González J. (2013). The impact of transposable elements in environmental adaptation. Mol. Ecol..

[9] McClintock B. (1984). The significance of responses of the genome to challenge. Science.

[10] Schultz D.C., Friedman J.R., Rauscher F.J. (2001). Targeting histone deacetylase complexes via KRAB-zinc finger proteins: The PHD and bromodomains of KAP-1 form a cooperative unit that recruits a novel isoform of the Mi-2a subunit of NuRD. Genes Dev..

[11] Carotti E., Carducci F., Greco S., Gerdol M., Di Marino D., Perta N., La Teana A., Canapa A., Barucca M., Biscotti M.A. (2022). Transcriptional contribution of transposable elements in relation to salinity conditions in teleosts and silencing mechanisms involved. Int. J. Mol. Sci..

[12] Czech B., Hannon G.J. (2016). One Loop to Rule Them All: The Ping-Pong Cycle and piRNA-Guided Silencing. Trends Biochem. Sci..

[13] Dechaud C., Volff J.N., Schartl M., Naville M. (2019). Sex and the TEs: Transposable elements in sexual development and function in animals. Mob. DNA.

[14] Jakymiw A., Lian S., Eystathioy T., Li S., Satoh M., Hamel J.C., Fritzler M.J., Chan E.K. (2005). Disruption of GW bodies impairs mammalian RNA interference. Nat. Cell. Biol..

[15] Liu J., Rivas F.V., Wohlschlegel J., Yates J.R., Parker R., Hannon G.J. (2005). A role for the P-body component GW182 in microRNA function. Nat. Cell. Biol..

[16] Meister G., Landthaler M., Peters L., Chen P.Y., Urlaub H., Lu¨hrmann R., Tuschl T. (2005). Identification of novel Argonaute-associated proteins. Curr. Biol..

[17] Chekulaeva M., Mathys H., Zipprich J.T., Attig J., Colic M., Parker R., Filipowicz W. (2011). miRNA repression involves GW182-mediated recruitment of CCR4-NOT through conserved W-containing motifs. Nat. Struct. Mol. Biol..

[18] Fabian M.R., Cieplak M.K., Frank F., Morita M., Green J., Srikumar T., Nagar B., Yamamoto T., Raught B., Duchaine T.F. (2011). miRNA-mediated deadenylation is orchestrated by GW182 through two conserved motifs that interact with CCR4-NOT. Nat. Struct. Mol. Biol..

[19] Grandbastien M.A., Audeon C., Bonnivard E., Casacuberta J.M., Chalhoub B., Costa A.P., Le Q.H., Melayah D., Petit M., Poncet C. (2005). Stress activation and genomic impact of Tnt1 retrotransposons in Solanaceae. Cytogenet. Genome Res..

[20] Hashida S.N., Kitamura K., Mikami T., Kishima Y. (2003). Temperature shift coordinately changes the activity and the methylation state of transposon Tam3 in *Antirrhinum majus*. Plant Physiol..

[21] Hashida S.N., Kishima Y., Mikami T. (2005). DNA methylation is not necessary for the inactivation of the Tam3 transposon at non-permissive temperature in *Antirrhinum*. J. Plant. Physiol..

[22] Hashida S.N., Uchiyama T., Martin C., Kishima Y., Sano Y., Mikami T. (2006). The temperature-dependent change in methylation of the *Antirrhinum* transposon Tam3 is controlled by the activity of its transposase. Plant Cell.

[23] Zeller G., Henz S.R., Widmer C.K., Sachsenberg T., Ratsch G., Weigel D., Laubinger S. (2009). Stress-induced changes in the Arabidopsis thaliana transcriptome analyzed using whole-genome tiling arrays. Plant J..

[24] Tittel-Elmer M., Bucher E., Broger L., Mathieu O., Paszkowski J., Vaillant I. (2010). Stress-induced activation of heterochromatic transcription. PLoS Genet..

[25] Fujino K., Hashida S.N., Ogawa T., Natsume T., Uchiyama T., Mikami T., Kishima Y. (2011). Temperature controls nuclear import of Tam3 transposase in *Antirrhinum*. Plant J..

[26] Makarevitch I., Waters A.J., West P.T., Stitzer M., Hirsch C.N., Ross-Ibarra J., Springer N.M. (2015). Transposable elements contribute to activation of maize genes in response to abiotic stress. PLoS Genet..

[27] Piacentini L., Fanti L., Specchia V., Bozzetti M.P., Berloco M., Palumbo G., Pimpinelli S. (2014). Transposons, environmental changes, and heritable induced phenotypic variability. Chromosoma.

[28] Feiner N. (2016). Accumulation of transposable elements in Hox gene clusters during adaptive radiation of Anolis lizards. Proc. Biol. Sci..

[29] Carducci F., Biscotti M.A., Forconi M., Barucca M., Canapa A. (2019). An intriguing relationship between teleost *Rex3* retroelement and environmental temperature. Biol. Lett..

[30] Carotti E., Carducci F., Canapa A., Barucca M., Greco S., Gerdol M., Biscotti M.A. (2021). Transposable elements and teleost migratory behaviour. Int. J. Mol. Sci..

[31] Rhee J.S., Choi B.S., Kim J., Kim B.M., Lee Y.M., Kim I.C., Kanamori A., Choi I.Y., Schartl M., Lee J.S. (2017). Diversity, distribution, and significance of transposable elements in the genome of the only selfing hermaphroditic vertebrate *Kryptolebias marmoratus*. Sci. Rep..

[32] Yuan Z., Liu S., Zhou T., Tian C., Bao L., Dunham R., Liu Z. (2018). Comparative genome analysis of 52 fish species suggests differential associations of repetitive elements with their living aquatic environments. BMC Genom..

[33] Auvinet J., Graça P., Ghigliotti L., Pisano E., Dettaï A., Ozouf-Costaz C., Higuet D. (2019). Insertion hot spots of DIRS1 retrotransposon and chromosomal diversifications among the Antarctic teleosts Nototheniidae. Int. J. Mol. Sci..

[34] Fry F.J., Rose A.H. (1967). Responses of vertebrate poikilotherms to temperature. Thermobiology.

[35] Huey R.B., Kingsolver J.G. (1989). Evolution of thermal sensitivity of ectotherm performance. Trends Ecol. Evol..

[36] Cossins A. (2012). Temperature Biology of Animals.

[37] Bernal M.A., Schmidt E., Donelson J.M., Munday P.L., Ravasi T. (2022). Molecular response of the brain to cross-generational warming in a coral reef fish. Front. Mar. Sci..

[38] Liu L., Zhang R., Wang X., Zhu H., Tian Z. (2020). Transcriptome analysis reveals molecular mechanisms responsive to acute cold stress in the tropical stenothermal fish tiger barb (*Puntius tetrazona*). BMC Genom..

[39] Manni M., Berkeley M.R., Seppey M., Zdobnov E.M. (2021). BUSCO: Assessing Genomic Data Quality and Beyond. Curr. Protoc..

[40] Altschul S.F., Gish W., Miller W., Myers E.W., Lipman D.J. (1990). Basic local alignment search tool. J. Mol. Biol..

[41] Biscotti M.A., Gerdol M., Canapa A., Forconi M., Olmo E., Pallavicini A., Barucca M., Schartl M. (2016). The lungfish transcriptome: A glimpse into molecular evolution events at the transition from water to land. Sci. Rep..

[42] Lisch D. (2013). How important are transposons for plant evolution?. Nat. Rev. Genet..

[43] Schrader L., Schmitz J. (2019). The impact of transposable elements in adaptive evolution. Mol Ecol..

[44] Naito K., Zhang F., Tsukiyama T., Saito H., Hancock C.N., Richardson A.O., Okumoto Y., Tanisaka T., Wessler S.R. (2009). Unexpected consequences of a sudden and massive transposon amplification on rice gene expression. Nature.

[45] Pecinka A., Dinh H.Q., Baubec T., Rosa M., Lettner N., Mittelsten Scheid O. (2010). Epigenetic regulation of repetitive elements is attenuated by prolonged heat stress in Arabidopsis. Plant Cell.

[46] Yasuda K., Ito M., Sugita T., Tsukiyama T., Saito H., Naito K., Teraishi M., Tanisaka T., Okumoto Y. (2013). Utilization of transposable element as a novel genetic tool for modification of the stress response in rice. Mol. Breed..

[47] Cavrak V.V., Lettner N., Jamge S., Kosarewicz A., Bayer L.M., Mittelsten Scheid O. (2014). How a retrotransposon exploits the plant’s heat stress response for its activation. PLoS Genet..

[48] Pappalardo A.M., Ferrito V., Biscotti M.A., Canapa A., Capriglione T. (2021). Transposable elements and stress in vertebrates: An overview. Int. J. Mol. Sci..

[49] Barik M., Bhattacharjee I., Ghosh A., Chandra G. (2018). Larvivorous potentiality of *Puntius tetrazona* and *Hyphessobrycon* rosaceus against *Culex vishnui* subgroup in laboratory and field based bioassay. BMC Res. Notes.

[50] Hunter R.G., Murakami G., Dewell S., Seligsohn M., Baker M.E., Datson N.A., McEwen B.S., Pfaff D.W. (2012). Acute stress and hippocampal histone H3 lysine 9 trimethylation, a retrotransposon silencing response. Proc. Natl. Acad. Sci. USA.

[51] Alberto-Payet F., Lassus R., Isla A., Daufresne M., Sentis A. (2022). Nine years of experimental warming did not influence the thermal sensitivity of metabolic rate in the medaka fish *Oryzias latipes*. Freshw. Biol..

[52] Horváth V., Merenciano M., González J. (2017). Revisiting the relationship between transposable elements and the eukaryotic stress response. Trends Genet..

[53] Zovoilis A., Cifuentes-Rojas C., Chu H.P., Hernandez A.J., Lee J.T. (2016). Destabilization of B2 RNA by EZH2 Activates the Stress Response. Cell.

[54] Slotkin R.K., Martienssen R. (2007). Transposable elements and the epigenetic regulation of the genome. Nat. Rev. Genet..

